# The Physiological and Molecular Links Between Physical Activity and Brain Health: A Review

**DOI:** 10.1177/26331055231191523

**Published:** 2023-08-17

**Authors:** Sarah Schock, Antoine Hakim

**Affiliations:** 1 Brain and Mind Research Institute, University of Ottawa, Ottawa, ON, Canada; 2 Division of Neurology, University of Ottawa, Ottawa, ON, Canada

**Keywords:** Aging, cognition, physical activity

## Abstract

There is currently an epidemic of sedentary behavior throughout the world, leading to negative impacts on physical health and contributing to both mortality and burden of disease. The consequences of this also impact the brain, where increased levels of cognitive decline are observed in individuals who are more sedentary. This review explores the physiological and molecular responses to our sedentary propensity, its contribution to several medical conditions and cognitive deficits, and the benefits of moderate levels of physical activity and exercise. Also presented is the recommended level of activity for overall physical health improvement.

## Introduction

Western society suffers from an epidemic of excessive sedentary behavior, defined as activities that do not markedly increase energy spending above the resting level.^1^ Using data from the 2015 National Health and Nutrition Examination Survey of the civilian US population, it was reported that 1 in 4 US adults sits for more than 8 hours a day.^2^ The SUNRISE study reported quantitatively assessed sedentary behavior in youth from 19 countries and showed that, on average, youth spent 56% of their time awake, amounting to 7.4 hours per day, being sedentary, with children from high income and upper-middle-income countries spending a greater amount of their day being sedentary.^3^ Prolonged sedentary behavior impacts multiple health domains. In 2010, the World Health Organization summarized the mortality and burden of disease attributable to selected major risks and estimated that 3.2 million people die per year due to physical inactivity, making sedentary behavior the fourth most important risk factor for mortality in the entire world accounting for 6% of all deaths.^4^

The COVID-19 pandemic had a significant impact on sedentary behavior. The review by Runacres et al^5^ showed that sedentary duration for children, defined as those younger than 18 years of age, increased by more than 2.5 hours per day, making them the most impacted group.

This review explores the physiological and molecular responses to our sedentary propensity, its contribution to several medical conditions and cognitive deficits, and the benefits of moderate levels of physical activity and exercise.

## Impacts of Sedentary Lifestyles

### On physiological and psychological functions relevant to brain health

In a systematic review of the literature, de Rezende et al^6^ reported strong evidence in youth of a connection between sedentary behavior and outcome of obesity, and some evidence for a negative impact of sedentary lifestyle on blood pressure, total cholesterol, self-esteem, and academic achievement. In the same publication, strong evidence was cited for a relationship in adults between inactivity and all-cause mortality, cardiovascular disease, type 2 diabetes, and metabolic syndrome. This study confirmed earlier data linking sedentary lifestyle with elevated triglycerides, high-density lipoprotein cholesterol and reduced insulin sensitivity.^7^ The rise in prevalence of diabetes mellitus with increasing sedentary time has also been documented in various studies.^8^ Park et al^9^ reviewed the potential health risks of sedentary lifestyle and detailed the extensive impact of physical inactivity on metabolic functions such as plasma triglycerides, cholesterol, and insulin sensitivity, resulting in significant health risks such as diabetes, hypertension, obesity, osteoporosis, and musculoskeletal diseases.

Women with the highest amount of inactivity were less likely to be normotensive and more likely to have elevated blood pressure and sstage II hypertension.^10^ Yang et al^11^ completed a systematic review of the relationship between sedentary behavior and sleep problems which allowed the conclusion that persistent sedentary behavior tended to be linked to an elevated risk for sleep problems such as insomnia and sleep disturbance.

The connection between sedentary behavior and depression is nuanced. While there is evidence that inactivity as a whole is associated with an increased risk of depression, subgroup analysis revealed that this relationship held only for intellectually passive sedentary behavior, such as watching television. If one is engaged in a mentally stimulating, but sedentary, activity such as video games, this was not correlated with risk of depression.^12^

### On inflammation

There is ample confirmatory evidence from the literature showing that sedentary behavior can create an inflammatory environment, and that physical activity can induce an anti-inflammatory environment. Considering all hypothetical extraneous factors, inactivity was linked with higher amounts of tumor necrosis factor alpha (TNFα) and leptin.^13^ Henson et al,^14^ demonstrated that the amount of time spent being inactive was negatively associated with levels of C-reactive protein (CRP), interleukin-6 (IL-6), and leptin. Parsons et al,^15^ confirmed that sedentary behaviors were linked with higher levels of IL-6, CRP, and tissue plasminogen activator (tPA). They also demonstrated that those who engaged in more physical activity had lower levels of these inflammatory mediators. A recent publication proposes that inflammation is the initial physiological trigger that limbs all harmful lifestyles to cognitive impairment.^16^

De Miguel et al^17^ reported that physical exercise increases complement cascade inhibitors, including clusterin, which binds to brain endothelial cells and decreases the expression of various neuroinflammatory genes. Participants with cognitive impairment took part in planned physical activities for 6 months. Results showed that this increased the plasma levels of clusterin, suggesting that exercise-induced anti-inflammatory elements are transferrable to the brain and target the cerebral vasculature.

### On cerebral blood flow (CBF)

CBF is closely matched to the brain’s metabolic demands through a number of mechanisms. Autoregulation matches the vascular response to changes in perfusion pressure, while vascular reactivity matches the caliber of blood vessels to vasoactive stimuli such as CO_2_. CBF also responds to neural activity through both neurovascular coupling and endothelium-dependent responses.^18^

There is also a multitude of evidence in the literature showing that persistent sitting is associated with a decline in cerebral perfusion. Carter et al,^19^ showed that when sitting is interrupted with 2 minutes of light walking every 30 minutes there is a significant decline in middle cerebral artery blood flow velocity compared to when sitting is uninterrupted. This gain was not observed if the time spent sitting was increased. Zlatar et al,^20^ also showed that more time spent inactive was linked to lower CBF in lateral and medial frontal regions, this is supported by initial studies by Bailey et al,^21^ which demonstrated that sedentary aging could cause a longitudinal decline in CBF.

### On cerebral structural integrity

White matter hyperintensities (WMH) are brain lesions that show up as areas of increased brightness on T2 weighted magnetic resonance imaging (MRI) and have been correlated with the risk of stroke and cognitive impairment.^22^ Brain WMH volume is increased with extended inactivity, and the correlation seems to be modulated by kidney function.^23^ Torres et al^24^ reported that more physical activity is correlated with less WMH. Rhyu et al,^25^ demonstrated in monkeys that physical activity increased the vascular volume fraction in the motor cortex. However, after a 3 month period of inactivity resulted in this benefit disappearing.

The link between sedentary behavior and brain atrophy is also clearly demonstrated in the literature. Siddarth et al,^26^ showed that medial temporal lobe, parahippocampal and entorhinal regions, and subiculum thickness was decreased depending on the number of hours per day 1 sat. In addition, Hashimoto et al,^27^ explored the link between hippocampal volume and physical activity and found that atrophy of this brain region was associated with sedentary behavior.

### On dementia risk

In 2011, Barnes et al,^28^ looked at the prevalence of cognitive impairment and its risk factors. They estimated that for North America, 21% of dementias could be caused by sedentary behavior. More recently, low, medium, and high fitness levels in Swedish women demonstrated that 32%, 25%, and 5%, subsequently developed dementia, respectively.^29^ Yan et al^30^ performed a systematic review and meta-analysis and found 18 applicable cohort studies comprising 250 063 participants, 2269 of those with dementia. Pooled results demonstrated that inactivity was significantly correlated with an increased risk of cognitive impairment (RR = 1.30; 95% CI: 1.12-1.51). Studies have shown that in hypertensive patients, incorporating regular physical activity can cause a regression or prevention of left ventricular hypertrophy.^31^

There is also a link between low muscles mass with cognitive decline. A cohort study of 8279 older adults, found that low muscle mass was correlated with an increase in pace and onset of executive function decline over the time period of 3 years.^32^

## Physiologic and molecular responses to exercise

### Physiologic responses to exercise

It has been known for decades that there is a potential protective effect of exercise in hypertension prevention. Paffenbarger et al,^33^ demonstrated that those who self-reported more than 5 hours per week of physical activity experienced a lower incidence of hypertension 2 to 3 decades later in life. The conclusions from 3 prospective studies, including the Nurses’ Health Study II, the Aerobics Center Longitudinal Study (ACLS), and the Coronary Artery Risk Development in Young Adults (CARDIA) study, showed that in those reporting physical activity, they have reduction in the incidence of the onset of hypertension.^34-36^ In addition, a study examining arterial stiffness in menopausal women showed that there were exercise-induced improvements over time and suggested this may indeed be a strategy to decrease cardiovascular disease risks in postmenopausal women.^37^ Physical activity is also known to reduce total cholesterol and triglyceride levels. It has been shown to increase high-density lipoprotein (HDL) cholesterol while holding low-density lipoprotein (LDL) steady and in some cases potentially offsetting increases in both LDL cholesterol and triglycerides. This occurs in a linear manner, with more activity causing a greater increase in HDL.^38^ A study analyzing the levels of triglycerides in patients with coronary heart disease showed that people how exercised showed a lower concentration of triglyceride levels.^39^

### Molecular responses to exercise

Chen and Nakagawa have recently reviewed the links between normal exercise physiology and the neurobiological mechanisms relevant to cognitive health that are associated with it.^40^

Physical activity can induce a wealth of beneficial molecular responses which have been shown to help with cell regeneration, appetite suppression, and protection against dementia among many others. It has been shown that *N*-lactoyl-phenylalanine, a signaling metabolite that suppresses feeding and obesity, is stimulated by exercise.^41^ Vivar et al^42^ have also recently reported that running keeps old adult-born neurons wired and prevents age-related memory loss.

Skeletal muscles can be viewed as an endocrine organ. They synthesize myokines such as irisin, and when activated they release these myokines into the circulation. Irisin contributes to glucose homeostasis, crosses the blood brain barrier, and results in the increased expression of BDNF in the hippocampus. The result is neuroprotection against memory decline.^43^

In post-stroke patients, acute effects of the intensity of physical activity on circulating molecules linked to neuroplasticity were increased. These included vascular-endothelial growth factor (VEGF) and insulin-like growth factor-1 (IGF1). In addition, blood lactate was able to facilitate the effect of high intensity exercise on BDNF and cortisol in a positive manner.^44^

### Reduction of inflammation

A study which reallocated 30 minutes of inactivity to medium and brisk physical activity was correlated with a positive inflammatory profile, made up of a higher adiponectin and lower complement component C3, leptin, and IL-6.^45^

In 2018, Tsai et al,^46^ demonstrated that a short bout of aerobic exercise caused serum levels of brain derived neurotrophic factor (BDNF) and insulin-like growth factor 1 (IGF-1) to increase. It is hypothesized that this increase may oppose the inflammatory environment. In an extensive review of the immune response to exercise, Scheffer and Latini^47^ confirmed that bouts of repeated physical exercise improve immunosurveillance and immunocompetence.

### Improved cerebral arterial and arteriolar functions

Aerobic exercise interventions have been demonstrated to improve vascular function in the large central arteries and in the peripheral circulation.^48^ Vicente-Campos et al,^49^ showed in 2012 that brain vasomotor reactivity improved with exercise, evidenced by higher blood flow velocity in the middle cerebral artery in response to hypercapnia.

Exercise activity also potentiated nitric oxide-dependent vascular reactivity^50^ and kept cerebral arterioles open. This was true even in the setting of chronic exposure to nicotine.^51^

### Improved white matter integrity

Aerobic Walking and social dance resulted in positive changes in the brain’s white matter.^52^ Burzynska et al,^53^ demonstrated that moderate to intense physical activity was linked to decreased white matter hyperintensity volume in healthy older adults. In addition, a randomized controlled trial in older women reported that resistance training decreased the progression of WMH.^54^ A review by Cooper et al,^55^ also detailed how running improved hippocampal neurogenesis, neural circuitry, and synaptic plasticity. The Reykjavik Study cohort is a sample from a larger cohort study, in this, Arnardottir et al,^56^ demonstrated that more gray and white matter volumes at baseline were correlated with an increase in exercise, and the 5-year change in MRI-derived white matter volume was correlated with total physical activity. The authors summarized their results by showing that a strong link existed between brain atrophy and sedentary behavior.

### Increased cerebral blood flow (CBF)

The literature has repeatedly confirmed the positive impact of regular physical activity on CBF.^57,58^ A long-term reduction in sedentary behavior increases peripheral vascular function and cerebral blood flow and may prevent impaired vascular function and reduced cerebral blood flow.^59^ One study measured global and regional cerebral blood flow before and after 20 minutes of cycling. The results demonstrated that CBF was improved by 10% to 12% in the hippocampus 15, 40, and 60 minutes after exercise cessation.^60^

Tomoto et al^61^ showed that 1 year of aerobic exercise decreased carotid arterial stiffness and improved cerebral blood flow, and the relationship held even in the setting of mild cognitive impairment.

### Better memory function

Sanders et al,^62^ used a systematic review and performed a meta-analysis to determine a dose-response relationship between physical activity and brain function. They confirmed results from a 10-year follow-up study by Hamer et al,^63^ which demonstrated that memory and executive function were protected by exercise.

Another systematic review in young to middle-aged adults showed that for those diagnosed with depression, some beneficial effect of exercise on memory was demonstrated.^64^ Also, in a study which investigated cycling as an intervention it was demonstrated that depressed individuals had a significantly higher post-intervention visuospatial memory function compared to the stretching control group.^65^ Therefore, exercise is beneficial throughout the lifetime to enhance and preserve memory function in individuals with and without chronic conditions.

More recently, a UK biobank prospective cohort study looked at the dose-response relationship between daily step count and amount and frequency of all-cause-dementia amongst adults. They found that a higher number of steps, and that just under 10 000 steps a day, was related with a lower risk of dementia. If these steps were done with higher intensity, the association was even stronger.^66^

One recent study also investigated how varying intensities of exercise could relate to distinct aspects of memory and mental health. Through self-reporting surveys on exercise and mental health along with memory tasks for short- and long-term memory, the investigators found that participants who had similar exercise regimes showed similar cognitive performance abilities. They also concluded that taking part in 1 type or intensity of exercise will not necessarily affect all areas of cognition or mental health equally.^67^

### Protection against medical and psychological conditions

Physical activity can impart protection against a number of medical and psychological conditions that impact large segments of Western society. A sample of these are listed below:

1. Obesity, type 2 diabetes, and several cardiometabolic disorders are reduced in response to an active lifestyle.^68-71^
2. Heart attack and stroke risk are reduced, and so is premature death.^72^
3. Exercise reduces anxiety.^73^
4. Physical activity offsets genetic risk for incident depression.^74^
5. Exercise results in a better night’s sleep.^75^
6. Reductions in upper respiratory infections.^76^
7. Many types of cancer are reduced with exercise.^66,77-79^

## Recommended Level of Activity

The Centers for Disease Control and Prevention recommend 150 minutes of moderate exercise per week. Examples given include brisk walking. The duration of the exercise can be shorter if the intensity of the aerobic activity is more vigorous.

1. For those over 60 years, the optimum step count is 6000 to 8000 a day.^80^
2. Exercise snacks add up.^72^ Total duration of physical activity—either long single or multiple shorter bouts of activity—is what provides the health benefits.^81^ Yoshida et al^82^ showed greater effects by performing a small number of contractions daily rather than a larger number of them once a week. Buffey et al,^83^ conducted a systematic review and meta-analysis of the literature and reported that frequent short interruptions of prolonged sitting with standing and light intensity walking improved biomarkers of cardiometabolic health.
3. Any activity is better than none, and the more active we are the more risks of chronic diseases drop.^84^
4. Excessive exercise can be harmful. In the extensive review of exercise-induced immune system response, Scheffer and Latini^47^ point out that levels of Neopterin and the risk of infection are elevated in those who practice strenuous acute exercise, reversing the benefits seen with light moderate exercise activity. Consequently, the level of neuroprotection is reduced, reversing the benefits seen with light, moderate, and regular training.
